# Editorial: Endocrine disruptors affecting human and companion animal endocrine function: similarities and indicators in the one health concept - volume II

**DOI:** 10.3389/fendo.2026.1837257

**Published:** 2026-04-14

**Authors:** Julianna Thuróczy, Caroline Serrano-Nascimento

**Affiliations:** 1 Animal Health Center Budafok, Gamma-Vet Ltd, Budapest, Hungary; 2 Molecular and Translational Endocrinology Laboratory (LEMT), Paulista School of Medicine (EPM), Federal University of São Paulo (UNIFESP), São Paulo, SP, Brazil; 3 Thyroid Physiology and Pathophysiology Laboratory (LaFFT), Institute of Environmental, Chemical and Pharmaceutical Sciences (ICAQF), Department of Biological Sciences, Federal University of São Paulo (UNIFESP), Diadema, SP, Brazil

**Keywords:** canine reproductive health, companion animals as sentinels, endocrine-disrupting chemicals (EDCs), environmental exposome, one health concept, translational toxicology

## Dogs at the frontline of endocrine disruption research – from semen to saliva in a one health framework

The growing body of research on endocrine-disrupting chemicals (EDCs) has steadily reshaped our understanding of environmental health risks across species. The second volume of *Endocrine Disruptors Affecting Human and Companion Animal Endocrine Function – Similarities and Indicators in the One Health Concept* underscores a critical paradigm shift: companion animals are not merely bystanders in environmental exposure scenarios but potential sentinels of shared chemical risk. As emphasized in the Research Topic introduction, environmental pollutants and persistent organic chemicals permeate ecosystems, bioaccumulate across food chains, and interfere with endocrine signaling in both humans and animals. Yet comparative endocrine toxicology remains insufficiently defined, particularly in identifying species-specific indicators of exposure and early dysfunction.

Within this context, two original investigations stand out for their methodological innovation and translational relevance: the analysis of per- and polyfluoroalkyl substances (PFAS) in dog serum in relation to semen quality and the exploration of dog saliva as a non-invasive matrix for chemical exposome studies. Together, these studies operationalize the One Health concept by positioning dogs as integrative biological indicators of environmental endocrine disruption.

## PFAS exposure and canine reproductive health

PFAS are among the most persistent and globally distributed synthetic pollutants. Their strong carbon–fluorine bonds confer resistance to degradation, enabling environmental persistence and bioaccumulation. While human epidemiological studies have linked PFAS exposure to endocrine disturbances, metabolic dysregulation, and impaired fertility, comparatively little is known about their effects in companion animals.

In the study of Weiss et al., a broad panel of 50 PFAS compounds was screened in serum of 65 Bernese Mountain Dogs, revealing detectable levels of multiple congeners in all samples Dominant compounds included PFOA, PFHxS, and PFOS, with concentrations comparable to those reported in humans and cats. This finding alone reinforces a key premise of the One Health model: domestic animals share environmental exposure pathways with their owners, particularly through indoor environments, food, and household materials.

Crucially, the study extended beyond exposure quantification to functional endocrine outcomes. Using detailed semen analysis—including sperm motility, morphology, and total sperm count—alongside endocrine biomarkers such as testosterone, anti-Müllerian hormone (AMH), and sex hormone-binding globulin (SHBG), the authors identified significant associations between PFAS concentrations and reproductive parameters. Notably, PFBS was positively associated with sperm motility and alterations in the free androgen index. While causality cannot be inferred, the integration of exposure assessment with endocrine and reproductive phenotyping marks a substantial methodological advance.

The evolutionary conservation of reproductive endocrine pathways across mammals lends translational weight to these findings. Dogs have relatively shorter lifespans and breed-specific genetic homogeneity, allowing detection of environmental effects within compressed timeframes. Observations of declining semen quality in dogs parallel reports in human males, raising the possibility that canine reproductive health may function as an early warning indicator of environmental fertility threats.

## Saliva as a window into the canine exposome

If serum-based monitoring identifies internal chemical burdens, the next frontier lies in developing scalable, minimally invasive tools for exposome assessment. The pilot study of Weiss et al. exploring dog saliva as an alternative matrix addresses precisely this challenge.

Traditionally, biomonitoring studies rely on blood sampling—a procedure that is invasive, resource-intensive, and often impractical for large-scale or repeated measurements. In contrast, saliva offers a non-invasive, stress-reducing, and cost-effective sampling method. In this study, paired saliva and serum samples from 30 privately owned dogs were analyzed for synthetic phenolic antioxidants (SPAs) and PFAS.

Fourteen SPAs and eight PFAS were detected in saliva and/or serum. Although concentrations were generally higher in serum, the presence of measurable levels in saliva confirms that oral fluid reflects systemic exposure. Importantly, none of the dogs were free of contaminants, and dominant compounds such as 2,4-di-tert-butylphenol and PFOS were frequently detected.

While no direct correlations between saliva and serum concentrations were established in this limited dataset, the proof-of-concept is significant. Saliva sampling could enable longitudinal exposure monitoring, early-life assessment, and broader population studies without the ethical and logistical constraints of blood collection. For veterinary medicine, this approach may facilitate routine screening of environmental exposures. For translational science, it opens possibilities for comparative human–animal exposome mapping within shared households.

## Dogs as translational sentinels

Taken together, these investigations exemplify the strategic value of companion animals in endocrine disruption research. The introduction to the Research Topic calls for deeper exploration of cross-species endocrine similarities and species-specific indicators of disease. The semen and saliva studies respond directly to this call by coupling exposure analytics with endocrine outcomes and by advancing methodological innovation in biomonitoring.

Dogs inhabit the same indoor environments, consume processed foods, and contact treated surfaces similar to their human counterparts. Yet their shorter lifespan and breed stratification may render them sensitive early detectors of cumulative chemical burden. Reproductive endpoints, in particular, offer measurable and biologically conserved markers of endocrine disruption.

The broader implication is clear: companion animals are not peripheral subjects but integral participants in environmental health surveillance. By integrating exposure chemistry, endocrine biomarkers, and minimally invasive sampling, the One Health framework moves from concept to practice.

Future research should expand cohort sizes, incorporate longitudinal designs, and refine saliva–serum calibration models. Ultimately, understanding endocrine disruption across species may not only improve veterinary outcomes but also sharpen our ability to detect and mitigate environmental risks to human health.

In an era of pervasive chemical exposure, dogs may be among our most informative—and most immediate—biological sentinels.

## From translational sentinels to human evidence

Beyond the identification of companion animals as environmental sentinels, which offer an innovative and highly informative perspective within the One Health framework, this Research Topic also discusses evidence on human health problems associated with exposure to widely recognized endocrine-disrupting chemicals. Thus, in addition to identifying novel biological indicators of exposure in dogs, the editorial includes studies addressing the effects of exposure to BPA and DDT and their associations with clinically relevant outcomes in humans. Taken together, these contributions highlight that endocrine disruption should be understood as a continuum, ranging from early detection in translational sentinel models to epidemiological and mechanistic evidence in human populations. By integrating these complementary perspectives, the Research Topic reinforces the relevance of both emerging biomarkers and human-centered evidence for understanding the full spectrum of health risks related to exposure to endocrine-disrupting chemicals across different stages of life.

## Bisphenol-A as a risk factor for infant neurodevelopment

Extending this comparative endocrine framework to early-life vulnerability, the review on prenatal exposure to Bisphenol A (BPA), conducted by Bello-Cortes et al. adds a crucial developmental dimension to the One Health narrative. BPA, a ubiquitous component of polycarbonate plastics and epoxy resins, has long been recognized as an endocrine-disrupting compound capable of mimicking or antagonizing estrogenic signaling, yet its implications for fetal neurodevelopment raise particularly urgent concerns.

Synthesizing human epidemiological evidence, the review reports that approximately 80% of analyzed studies identified associations between maternal BPA exposure during pregnancy and behavioral alterations in offspring, with male infants appearing especially vulnerable during third-trimester exposure windows. Mechanistically, BPA’s ability to bind estrogen receptors (ERα, ERβ, and with even higher affinity ERRγ), interfere with androgen signaling, and induce epigenetic modifications provides biological plausibility for long-term neurodevelopmental programming effects. Importantly, BPA can be deconjugated in the placenta, reactivating free BPA and facilitating fetal exposure at developmental stages characterized by limited detoxification capacity.

The review further highlights the relevance of non-monotonic dose–response relationships, challenging traditional toxicological assumptions that lower exposure necessarily equates to lower risk. Within the Developmental Origins of Health and Disease (DOHaD) framework, prenatal BPA exposure may “program” susceptibility to later neurobehavioral disorders, reinforcing the concept that endocrine disruption during critical windows exerts lifelong consequences.

When considered alongside findings in dogs demonstrating endocrine and reproductive sensitivity to environmental contaminants, the BPA literature underscores that endocrine vulnerability begins before birth and transcends species boundaries. Companion animals and humans share indoor environments where BPA-containing materials remain prevalent, strengthening the translational relevance of cross-species exposure assessment. Integrating developmental neurotoxicity into the One Health discourse thus broadens the sentinel model: endocrine disruption is not confined to adult metabolic or reproductive endpoints but may shape neurodevelopmental trajectories from the earliest stages of life.

## Metabolic consequences of DDT exposure in humans

Dichlorodiphenyltrichloroethane (DDT) is a persistent organochlorine pesticide whose use has been restricted in many countries, but both the parent compound and its metabolites, particularly DDE and DDD, remain widespread in the environment because of their high stability and bioaccumulative properties. Their continued detection in biological samples and their association with endocrine and metabolic alterations make them important contaminants in the study of human health effects.

A systematic review and meta-analysis conducted by Tian and colleagues extends the discussion toward metabolic disease by examining the association between DDT exposure and type 2 diabetes mellitus (T2DM). By integrating 13 epidemiological studies, the authors reported a significant positive association between DDT or its byproducts and T2DM risk, with an overall pooled odds ratio of 1.13 (95% CI: 1.08–1.15). Subgroup analysis identified p,p′-DDE as a major source of heterogeneity and as a particularly relevant biomarker associated with diabetes risk. Although publication bias and small-study effects were detected, the authors concluded that the overall impact on the results was limited, supporting the robustness of the association. These findings broaden the scope of endocrine disruption research by reinforcing the idea that persistent environmental contaminants may contribute not only to reproductive and developmental abnormalities but also to the growing burden of metabolic disease.

As noted by the authors, these findings should be interpreted in light of some inherent limitations, including variability across the study designs employed, the methods used for exposure assessment, and the challenges of fully addressing co-exposures and complex dose-response relationships. In addition, both humans and other animals are exposed throughout life, and during different temporal windows, to multiple endocrine disruptors, which makes it difficult to isolate the effect of a single compound and compromises the establishment of cause-and-effect relationships across different studies in the literature.

## From population-based evidence to *in vitro* investigation

Although epidemiological studies are essential for identifying associations between environmental contaminants and disease risk in human populations, mechanistic investigations are equally important for clarifying the biological pathways that may underlie these observations. In addition, these studies allow the identification of new substances with potential endocrine-disrupting activity in the organism. In this context, *in vivo* and *in vitro* experimental models provide a critical complementary perspective by helping to establish the molecular and cellular mechanisms involved in the disruption of homeostasis. This transition from population-level evidence to mechanistic understanding strengthens the interpretation of endocrine disruption as a complex and multisystem process, linking the observed health outcomes to their associated biological mechanisms.

The study of Pastorino et al., investigating the effects of chlorpyrifos (CPF), a widely used organophosphate pesticide extensively applied in agriculture and pest control, on hypothalamic pathways regulating energy balance offers relevant mechanistic evidence that this compound may interfere with central neuroendocrine circuits involved in metabolic control. Using murine hypothalamic cells and chronically exposed mice, the authors showed that CPF was associated with increased orexigenic signaling, as reflected by higher *Npy* and *Agrp* expression *in vitro* and *in vivo*, as well as enhanced neuropeptide secretion *in vitro*. Importantly, these effects appeared to be mediated, at least in part, by estrogen receptor beta, since CPF increased the ERβ/ERα ratio and pharmacological inhibition of ERβ attenuated the induction of these neuropeptides. The study also showed increased leptin receptor expression, suggesting broader disruption of hypothalamic pathways involved in energy homeostasis. Taken together, these findings support the view that CPF may act as a metabolic disruptor at the central neuroendocrine level, broadening the endocrine disruption framework to include interference with hypothalamic regulation of feeding behavior and metabolic balance.

Collectively, the articles included in this Research Topic reinforce endocrine disruption as a multisystem and translational phenomenon that spans species, developmental windows, and multiple levels of biological organization. By demonstrating that shared environmental exposures can affect both humans and other animals through interconnected biological pathways, these studies further strengthen the One Health concept and emphasize the close interdependence among environmental, veterinary, and human health. Finally, the Associate Editors would like to thank the authors, reviewers, and the Frontiers editorial team for their trust and for their outstanding work on this Special Issue.

