# Correction: Editorial: Endocrine disruptors affecting human and companion animal endocrine function: similarities and indicators in the one health concept - volume II

**DOI:** 10.3389/fendo.2026.1856379

**Published:** 2026-04-20

**Authors:** Frontiers Production Office

**Affiliations:** Frontiers Media SA, Lausanne, Switzerland

**Keywords:** canine reproductive health, companion animals as sentinels, endocrine-disrupting chemicals (EDCs), environmental exposome, one health concept, translational toxicology

Affiliation “Animal Health Center Budafok, Gamma-Vet Ltd, Budapest, Hungary” was omitted for author Julianna Thuróczy. This affiliation has now been added for author Julianna Thuróczy.

Author Julianna Thuróczy was erroneously assigned to affiliation “Molecular and Translational Endocrinology Laboratory (LEMT), Paulista School of Medicine (EPM), Federal University of São Paulo (UNIFESP), São Paulo, SP, Brazil”. This affiliation has now been removed for author Julianna Thuróczy.

The original version of this article has been updated.

